# A new perspective on cancer treatment: the interaction and application prospect between ICIs and radiotherapy

**DOI:** 10.1002/pro6.70047

**Published:** 2026-01-11

**Authors:** Guiling Song, Lei Wang, Guopeng Zhao, Song Tan, Xin Liu, Xinghua Li

**Affiliations:** ^1^ Department of Chemical medicine Yantai Center For Food And Drug Control Yantai Shandong China; ^2^ Department of Oncology Yantai Hospital of Traditional Chinese Medicine Yantai Shandong China; ^3^ Peninsula Cancer Research Center Binzhou Medical University Yantai P.R. China

**Keywords:** Clinical application, ICIs, Radiotherapy, Synergistic mechanism

## Abstract

The interaction between immune checkpoint inhibitors (ICIs) and radiotherapy has significantly improved the treatment outcomes for patients with cancer. ICIs activate the immune system by blocking the programmed death‐1 (PD‐1), programmed cell death‐Ligand 1(PD‐L1), and cytotoxic T‐lymphocyte‐associated protein 4 (CTLA‐4) signaling pathways, thereby killing tumor cells. Radiotherapy activates local and abscopal effects by killing tumor cells, inducing immunogenic death, and releasing antigens and damage‐related molecules. The combination of the two methods forms a closed‐loop process of “antigen release‐immune activation,” which significantly improves the therapeutic effect of patients with cancer. Clinical studies have shown that ICIs combined with radiotherapy can significantly improve the objective response rate (ORR) and progression‐free survival (PFS) in patients with solid tumors, such as Non‐Small Cell Lung Cancer (NSCLC) and melanoma. However, heterogeneous responses, immune‐related adverse events (irAEs), and drug resistance are still common clinical problems. Optimizing the fractionation of radiotherapy dose, timing of synchronous intervention, and biomarkers, such as tumor mutation burden, are key to improving tumor treatment efficacy. In the future, precise tumor treatment is expected to improve patient prognosis through the interaction between ICIs and radiotherapy.

## Graphical abstract

The combination of immune checkpoint inhibitors (ICIs) and radiotherapy synergistically enhances efficacy through an “antigen release‐immune activation” closed‐loop mechanism, significantly improving outcomes in solid tumors (e.g., lung cancer, melanoma). However, challenges remain, including heterogeneous efficacy, adverse reactions, and drug resistance.

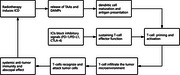

Currently, the treatment for malignant tumors primarily includes drug therapy, surgical resection, and radiotherapy. While killing tumor cells, traditional drug therapy can also cause damage to normal cells, leading to serious adverse reactions such as neutropenia, anemia, alopecia, nausea, vomiting, and oral mucositis. Therefore, the selection of higher efficacy and better safety in clinical treatment has become the premise of current tumor treatment. Recently, among numerous anti‐tumor drugs, ICIs have attracted much attention. ICIs mainly include PD‐1 inhibitors, PD‐L1 inhibitors, and CTLA‐4 inhibitors.^1^

By suppressing T cells and activating immune system responses, ICIs have increased the 5‐year survival rate of patients with solid tumors such as melanoma and lung cancer by 20%‐30%.^2^ Radiotherapy kills tumor cells using ionizing radiation while inducing immunogenic cell death and activating local and abscopal effects. The combination of the two forms a closed‐loop process of “antigen release‐immune activation,” which significantly improves the therapeutic effect of patients with cancer.^3^, ^4^ In this closed‐loop process, radiotherapy induces immunogenic cell death and releases tumor antigens and damage‐associated molecular patterns (DAMPs), promoting dendritic cell maturation and antigen presentation. This primes T cell activation, whereas ICIs block inhibitory checkpoints to sustain T cell effector function, thereby amplifying systemic anti‐tumor immunity (Figure 1).^5^ In animal experiments, ICIs combined with radiotherapy can significantly shrink tumor cells; however, there are significant differences in the clinical treatment effects.^6^ In this study, the mechanism of the interaction between ICIs and radiotherapy is discussed, the advantages and challenges of ICIs combined with clinical research data are analyzed, and the application prospects of combined therapy between ICIs and radiotherapy are discussed.

**FIGURE 1:**
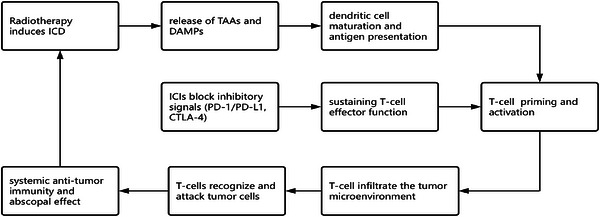
Schematic illustration of the “antigen release‐immune activation” closed‐loop mechanism synergized by radiotherapy and ICIs.

## 1 SYNERGISTIC MECHANISMS OF ICIS AND RADIOTHERAPY

### 1.1 Immune regulatory mechanisms of ICIs

#### 1.1.1 Mechanisms of action of PD‐1/PD‐L1 antibodies

When T cells detect antigens presented by major histocompatibility complex (MHC) molecules on tumor cells, they activate themselves and release cytokines.^7^ If T cells are active at all times, they increase surface PD‐1 protein levels. Simultaneously, tumor cells begin to express PD‐L1 under the influence of signals released by T cells. When PD‐1 on the surface of T cells binds to PD‐L1 on the surface of tumor cells, it will cause the signal transduction function of T cells to be weakened. Consequently, the efficacy of T cells is reduced, the proliferation rate is reduced, and cell apoptosis may occur, thereby assisting tumor cells to evade surveillance by the immune system.^8^ PD‐1 antibody can block PD‐1 on T cells, whereas PD‐L1 antibody can block PD‐L1 on tumor cells, relieve the inhibitory effect of T cells, and restore anti‐tumor activity, thereby effectively killing tumor cells.^9^

#### 1.1.2 Mechanisms of action of CTLA‐4 inhibitors​

In the process of T cell activation, antigen‐presenting cells (APC) activate T cells through the MHC‐antigen peptide‐T cell receptor (TCR) and B7‐CD28 dual signaling system. Activated T cells upregulate the expression of CTLA‐4 and block the CD28‐mediated PI3K‐AKT‐mTOR signaling pathway through competitive binding of CTLA‐4 and CD28 to B7 molecules (CD80/CD86) on the surface of APC. Simultaneously, it can also recruit SHP‐2 phosphatase through the immunoreceptor tyrosine inhibitory motif (ITIM) of CTLA‐4 intracellular domain to dephosphorylate the TCR signaling pathway and inhibit TCR signaling cascade amplification via immunoreceptor tyrosine ITIM.^10^ In the tumor microenvironment, tumor cells secrete TGF‐β to induce Tregs to highly express CTLA‐4. Tregs bind CTLA‐4 to B7 molecules, trigger endocytosis, actively internalize and degrade B7 molecules on APC surface, and generate CD8+ T cells in the anergic state. In this process, the IL‐10 secretion capacity of Tregs is enhanced, and effector T cell function is further inhibited.^11^

#### 1.1.3 Mechanisms of action of other ICIs​

Emerging immune checkpoints such as LAG‐3, TIM‐3, and TIGIT are also being investigated in combination with radiotherapy.^12^, ^13^, ^14^ For instance, LAG‐3 inhibition may reverse T cell exhaustion exacerbated by radiation, whereas TIM‐3 blockade enhances dendritic cell function.^15^ Although clinical evidence is still emerging, these targets represent promising avenues for overcoming resistance to PD‐1/CTLA‐4 blockade in combination with radiotherapy.

### 1.2 Immunomodulatory effects of radiotherapy

#### 1.2.1 It acts directly on tumor cells

Radiotherapy uses ionizing radiation to induce DNA double‐strand breaks (DSBS) in tumor cells, leading to cessation of cell cycle progression and cell apoptosis. In this process, the release of tumor‐associated antigens (TAA) and damage signals provides a material basis for the subsequent immune response.^16^ Furthermore, radiotherapy enhances the expression of MHC‐I molecules, therefore increasing their likelihood of being recognized by T cells.^17^

#### 1.2.2 Activation of innate immune signaling pathways​​

Radiation‐induced DNA damage can activate the cGAS‐STING signal transduction pathway in tumor cells and promote the secretion of type I interferon (IFN‐α/β). This process enhances maturation and antigen presentation by dendritic cells (DCS).^18^ Concurrently, released DAMPs activate innate immune cells by binding to pattern recognition receptors such as TLR4 and can also promote the assembly of inflammasome complexes such as NLRP3, thereby amplifying inflammatory signals.^19^

#### 1.2.3 Remodeling the tumor microenvironment

Radiotherapy can reduce the infiltration of immunosuppressive cells such as Tregs and myeloid‐derived suppressor cells (MDSCs) into the tumor microenvironment. It also reduced the secretion of immunosuppressive factors such as TGF‐β and IL‐10.^20^ Additionally, radiotherapy can promote the normalization of blood vessels and improve the hypoxic microenvironment of tumors, thereby enhancing the infiltration and function of effector T and natural killer (NK) cells.^21^

#### 1.2.4 Induction of abscopal effects

Local radiotherapy can trigger tumor shrinkage at distant sites through systemic immune activation. The abscopal effect refers to the regression of non‐irradiated tumors following local radiotherapy mediated by systemic immune activation. This phenomenon relies on the release of tumor antigens, which are taken up by DCS, leading to T cell priming and migration to distant sites.^22^ This process often requires a combination of ICIs to overcome the immune escape of distant tumors.

### 1.3 Interaction between ICIs and radiotherapy

The timing and dose fractionation of combination therapy critically influence treatment outcomes. Preclinical studies have shown that the administration of PD‐1 antibody within 24–72 h after radiotherapy maximizes the expansion of T cells, which may be related to the time window of radiation‐induced antigen exposure and immune cells.^23^ In terms of dose fractionation, hypofractionation (8 Gy×3 fractions) was more effective than conventional fractionated radiotherapy (2 Gy×25 fractions) in activating the cGAS‐STING signaling pathway, thereby promoting interferon secretion and CD8 + T cell infiltration,^24^ as shown in Table 1. Furthermore, the selection of the radiotherapy target volume affects the immune response. Irradiation of primary tumors with strong immunogenicity can significantly improve the regression rate of distant metastases.^25^

**TABLE 1:** Immune activation effects of different radiotherapy fractionation regimens.

Mode of segmentation	Dose	Mechanisms of immune activation	Infiltration of CD8+ T cells	Incidence of abscopal effects
hypofractionation	8 Gy×3 fractions	Potent activation of the cGAS‐STING pathway	Significant increase	28%
Conventionally fractionated radiotherapy	2 Gy×25 fractions	Mild activation of innate immune signaling	Moderate increase	10%‐15%

From the perspective of the tumor microenvironment, radiotherapy reduces the infiltration of Tregs and MDSCs by reducing the expression of immunosuppressive factors such as transforming growth factor (TGF‐β) and IL‐10, improving ICI efficacy.^26^ A combination of PD‐1 inhibitors and stereotactic body radiation therapy (SBRT) resulted in an objective response rate (ORR) of 58% in patients with Non‐Small Cell Lung Cancer (NSCLC), exceeding the effect of PD‐1 blockade alone.^27^ Despite this, inconsistencies in efficacy still exist, which may be related to the inhomogeneity of the radiation dose distribution and the diversity of human leukocyte antigen (HLA) molecules in patients.^28^

Notably, combination therapies have the potential to exacerbate adverse immune effects. The research showed that grade ≥3 pneumonitis occurred in 18% of patients who received a CTLA‐4 inhibitor plus lung radiation therapy, a rate that was significantly higher than that among patients who received ICIs alone.^29^ Therefore, individualized treatment strategies for these biomarkers are important. Presently, the tumor mutation burden, PD‐L1 expression level, and proportion of CD8+Ki67+T cells in peripheral blood have been proven to be effective indicators for predicting the response to combination therapy.^30^ Future studies are needed to further explore the interaction mechanism between radiotherapy and ICIs and optimize the treatment sequence through multi‐omics analysis to improve the clinical treatment effects.^31^

## 2 CLINICAL TRANSLATION ACROSS SOLID TUMORS

ICIs' combination and radiotherapy have transitioned from exploratory trials to established clinical practice in certain settings, such as unresectable NSCLC and melanoma. However, many applications remain under investigation in Phase II and III trials. The key clinical evidence, highlighting the study design, outcomes, and limitations, is summarized below.

### 2.1 Breakthrough evidence for lung cancer treatment

Clinical studies on NSCLC have provided strong support for the synergistic treatment with ICIs combined with radiotherapy. As a phase II non‐randomized controlled trial, the DOLPHIN study^32^ explored a new model of immunotherapy combined with radiotherapy, followed by sequential maintenance therapy. This study enrolled patients with unresectable locally advanced NSCLC who were treated with duvalumab during concurrent chemoradiotherapy (cCRT) and transitioned to single‐agent maintenance. The latest data showed that the 1‐year PFS rate of this regimen was 72.1% (90%CI 59.1%‐85.1%), the mPFS was 25.6 months, and the ORR was 90.9% (95%CI 75.7%‐98.1%). PACIFIC (NCT02125461)^33^ is a global, randomized, double‐blind, placebo‐controlled phase III clinical trial. To evaluate the efficacy of the PD‐L1 inhibitor durvalumab as a consolidation therapy after cCRT in patients with unresectable stage III NSCLC. This study establishes “cCRT plus Durvalumab consolidation” as the standard of care in this patient population. This study's results showed that patients who received Durvalumab had better median progression‐free survival (mPFS: 16.9 months vs. 5.6 months, hazard ratio HR = 0.52) and overall survival (mOS: 47.5 vs. 29.1 months, HR = 0.73), and the safety profile was manageable. The PACIFIC regimen effectively utilizes the closed‐loop mechanism of “antigen release‐immune activation” by inducing local immunogenic cell death to release antigen, followed by Durvalumab to maintain T cell activity, and provides significant survival benefits for patients with locally advanced NSCLC. This marks a model for the successful combination of ICI therapy and RT.

For patients with positive driver genes, preclinical studies have found that the third‐generation epidermal growth factor receptor‐tyrosine kinase inhibitor (EGFR‐TKI) osimertinib can induce immunogenic cell death by inhibiting the downstream signaling pathway of EGFR and cooperating with radiotherapy, while reducing vascular endothelial growth factor (VEGF) levels to improve tumor vascular normalization and increase CD8+T cell infiltration density by 4.2 times.^34^ Phase III data from the SINDAS trial (NCT03391869)^35^ further support this theory: The mPFS of patients with EGFR‐mutated oligometastatic NSCLC treated with concurrent SBRT with osimertinib reached 20.2 months (vs 12.5 months with TKI alone, HR = 0.62), and the 3‐year OS rate increased to 48%, which provides high‐level evidence for the “target‐radiotherapy‐immunotherapy” triple therapy mode.

### 2.2 Research progress in colorectal cancer

The clinical practice of colorectal cancer provides an important direction for radiotherapy combined with immunotherapy. Clinical studies have shown that conventional radiotherapy doses can directly cause the death of effector immune cells in irradiated areas. Radiotherapy can also promote the expression of TAA and stimulate immune responses, thereby altering the immune microenvironment.^36^ Furthermore, radiotherapy can enhance tumor cell recognition by the immune system, change the immune properties of tumors, and enhance the immune response.^37^ Kang et al.^38^ revealed that the expression of PD‐L1 and IFN‐γ was upregulated in tumor tissues of patients with locally advanced colorectal cancer after neoadjuvant chemoradiotherapy, and that these expressions were closely related to patients' prognosis. These findings provide theoretical support for combining neoadjuvant chemoradiotherapy with ICIs. To date, several clinical trials (NCT04109755, NCT02586610, NCT02921256, and NCT03104439) are ongoing to evaluate the safety and efficacy of ICIs and radiotherapy in colorectal cancer treatment. These study results are expected to provide an important reference for the clinical application of radiotherapy in combination with ICIs therapy.

### 2.3 Research progress of liver cancer

A significant synergistic effect was observed when SBRT was combined with the ICIs. This combination therapy significantly prolonged the mPFS and mOS in patients with hepatocellular carcinoma (HCC). The irAEs associated with this treatment were manageable, indicating its safety profile. In one study,^39^ TACE, SBRT, and avelumab were sequentially administered to 33 patients with advanced HCC. The results showed that 14 patients achieved complete remission after treatment. Other studies have found that high levels of PD‐L1 in the serum are associated with tumor aggressiveness and poor prognosis. After radiotherapy, the expression of PD‐L1 in tumor cells is upregulated in patients with advanced HCC, leading to a significant increase in serum PD‐L1 levels. Although this may increase the immune escape ability of the tumor, when combined with ICIs, it can have a synergistic effect, thereby enhancing ICIs' efficacy and providing greater clinical benefit to patients.

### 2.4 Research progress of melanoma

ICIs’ combination and radiotherapy has shown synergistic effects in the treatment of melanoma. Radiotherapy can promote the infiltration of CD8+T cells and enhance the expression of immune cytokines, thereby regulating the tumor microenvironment.^40^ A clinical study involving 24 patients with locally advanced melanoma was designed to compare preoperative ipilimumab plus radiotherapy with postoperative adjuvant therapy.^41^ An overall ORR of 64% and DCR of 73% were significantly better with neoadjuvant therapy than with ipilimumab alone. Furthermore, preoperative neoadjuvant CTLA‐4 inhibitors combined with radiotherapy showed good efficacy without grade 3 adverse events.

To our knowledge, there are currently no unified treatment guidelines for oligoprogressive melanoma. A retrospective analysis reveals^42^ that 68% of the patients with melanoma opted for oligoprogressive disease surgery after ICIs therapy. Of these patients, 76% showed no evidence of disease, suggesting that surgery may have benefited these patients. Stereotactic radiotherapy and stereotactic body ablative radiotherapy, either alone or in combination with ICIs, can improve the PRR, induce long‐term disease control, and prolong PFS in patients with melanoma who develop oligoprogressive progression after ICIs treatment.^43^ Recent studies have shown that local radiotherapy can benefit patients with oligometastatic melanoma after ICIs treatment, especially those with a good local response and high expression of PD‐L1.^44^

### 2.5 Advances in the treatment of other solid tumors

Besides the abovementioned tumors, the combination of radiotherapy has shown unique potential in the treatment of other solid tumors, and this study's findings have brought a new dawn in cancer treatment (Table 2). This is particularly true in patients with HER2‐negative triple‐negative breast cancer. A phase II study demonstrated significant improvement in efficacy when the PD‐1 inhibitor pembrolizumab was combined with radiation therapy.^45^ Combination therapy resulted in a significant increase in the density and an ORR of 41%, which was significantly higher than that observed with pembrolizumab alone. From a mechanistic perspective, radiotherapy can promote the migration of CD8+ T cells to the tumor microenvironment, and ICIs exert a significant synergistic effect by blocking PD‐L1‐mediated immunosuppression.^46^

**TABLE 2:** Clinical outcomes of ICIs combined with radiotherapy in solid tumors.

Categories	Clinical Research	Treatment Options	Study Design	Research Results	Limitayions
NSCLC	DOLPHIN study^[^ ^33^ ^]^	Duvilumab + radiotherapy, duvilumab maintenance therapy.	Phase II, multicenter, single‐arm	The ORR was 70%	Small sample size; non‐randomized controlled design; short follow‐up time; high incidence of adverse events at grade 3 and above; no control group.
NSCLC	NCT02125461^[^ ^34^ ^]^	cCRT+duvalumab	Randomized;Double‐blind;multicenter;Phase III;	mPFS: 16.9m (vs 5.6m placebo), mOS: 47.5m (vs 29.1m placebo); 5‐yr OS rate: 42.9%	PD‐L1 selection debated; long‐term toxicity data ongoing; limited data on the impact of specific radiotherapy parameters.
NSCLC	SINDAS^[^ ^35^ ^]^	Osimertinib + radiotherapy	Phase III;multicenter;	mPFS reached 20.2 months (vs the TKI‐only group 12.5 months), and the 3‐year OS rate increased to 48%.	Short follow‐up time; small sample size; uneven distribution of metastatic lesions; unclear resistance mechanisms.
Colorectal cancer	NCT02837263	Pembrolizumab+SBRT	Phase 1;Single‐center;single‐arm;	No Results Posted	Lack of a control group; small sample size; single‐center design; no blinded design adopted.
Colorectal cancer	NCT02437071	Pembrolizumab+radiotherapy	Phase II;single‐arm;Single‐center;	No Results Posted	Lack of a control group, small sample size, and single‐center design.
HCC	START‐FIT^[^ ^39^ ^]^	TACE+SBRT+avelumab	Phase II;single‐arm;multicenter,	The ORR is 67%, the CRR is 42%, the PRR is 24%, and the DCR is 70%.	Lack of a control group; small sample size; insufficient representativeness of the patient population; lack of long‐term follow‐up data.
Melanoma	TACTICS‐HCC study^[^ ^41^ ^]^	TACE+PD‐L1 inhibitors + Radiotherapy	Phase II, Single‐center、single‐arm;	The ORR rate of patients in the neoadjuvant therapy group reached 64%, with a DCR of 73%, and a 2‐year PFS of 73%.	Small sample size; non‐randomized design; inconsistent surgical standards; high patient heterogeneity.
Triple‐negative Breast Cancer	NCT03366844^[^ ^45^ ^]^	Pembrolizumab + radiotherapy	Phase II;single‐arm;multicenter,;	The ORR was 41% and the density of TILs increased	Lack of control group; small sample size; single‐center design; high patient heterogeneity.

It has also entered an exploratory stage of esophageal cancer treatment. In the KEYNOTE‐975 study, neoadjuvant treatment with DS‐8201 plus pembrolizumab for HER2‐positive esophageal cancer resulted in a pCR rate of 45%. The timing of synergistic mechanisms with radiotherapy has also entered the clinical exploration stage.^47^ This study not only provides novel therapeutic options for patients with esophageal cancer but also underscores the potential of combining immunotherapy and radiotherapy.

## 3 CURRENT CHALLENGES AND MECHANISMS OF RESISTANCE

ICI combination and radiotherapy provide a new direction to break through the bottleneck of traditional cancer treatment through the closed‐loop mechanism of “antigen releasing‐immune activation.” Radiation‐induced immunogenic cell death releases tumor antigens and activates innate immune signals, such as the cGAS‐STING pathway, whereas ICIs relieve T‐cell suppression and amplify systemic antitumor responses. Clinical studies have shown that combination therapy significantly improves the ORR and survival benefit of patients with cancers such as NSCLC and melanoma. However, the heterogeneity in efficacy, irAEs, and drug resistance remains a major challenges.^48^, ^49^, ^50^

The core of drug resistance lies in the dynamic escape mechanisms of tumor immunoediting. Following radiotherapy, residual tumor cells exhibit a simplified antigenic landscape, evading T‐cell recognition by downregulating HLA‐I molecules or losing β2‐microglobulin. Concurrently, clones with a profibrotic phenotype were selected for immune pressure.^51^ These clones recruit CAFs via the CXCL12/CXCR4 axis, constructing a physical barrier that impedes T‐cell infiltration. Counterstrategies primarily focus on three aspects: Utilizing epigenetic modulators (DNMT inhibitors) to induce the re‐expression of silenced neoantigens;^52^ Designing personalized vaccines targeting the evolutionary trajectory of neoantigens post‐radiotherapy;^53^ Targeting PAI‐1 secreted by CAFs to reverse the fibrotic microenvironment. Recent single‐cell sequencing studies further elucidate that the proportion of TCF‐1+ stem‐like CD8+ T cells post‐radiotherapy determines the strength of immune memory, while the expansion of the PD‐1+Tim‐3+ terminally exhausted subset predicts the risk of recurrence. This study identified new biomarkers for dynamic monitoring.^54^

Emerging evidence also highlights the roles of cancer stem cells (CSCs) and gut microbiota in driving resistance to radiotherapy. CSCs, with their self‐renewal capacity and enhanced DNA repair mechanisms, can survive radiotherapy and repopulate tumors, often exhibiting an immunosuppressive phenotype.^55^ Gut microbiota modulates systemic immune responses and influences ICIs' efficacy. Specific bacterial species are associated with improved treatment outcomes, and dysbiosis may contribute to resistance.^56^ Incorporating strategies to target CSCs or modulate the gut microbiome is a promising strategy for overcoming resistance to combination therapy.

In terms of safety, the superposition of radiotherapy and ICIs may aggravate irAEs, such as pneumonia and colitis. Therefore, individualized dose optimization was performed to minimize damage to normal tissue. Combination with immunomodulators can help balance therapeutic effects and toxicity. Furthermore, it is of great significance to explore the feasibility of radiotherapy combined with targeted immune triple modality therapy for driver gene‐positive tumors. This innovative strategy is expected to overcome the limitations of conventional therapies and provide patients with more effective treatment options.^57^

## 4 FUTURE PERSPECTIVES AND BIOMARKER‐DRIVEN STRATEGIES

Future perspectives on ICI combination and radiotherapy will hinge on optimizing combination strategies and refining biomarker‐driven patient selection. Beyond the established synergy, novel approaches such as integrating radiotherapy with bispecific antibodies, adoptive cell therapies, or targeted radiosensitizers are under investigation to amplify anti‐tumor immunity and overcome resistance mechanisms. Personalized radiotherapy schedules tailored to a patient's immune status and tumor phenotype are crucial.

The main factors influencing the synergistic effects of ICIs and radiotherapy in cancer treatment are the dose and timing of radiotherapy. In the treatment scheme of high‐dose fractionated radiotherapy, due to its efficient STING pathway activation ability, it shows a better therapeutic effect than conventional fractionated radiotherapy. Compared to sequential application, the simultaneous application of radiotherapy and ICIs can maximize the time of antigen exposure and T‐cell activation, thereby further improving the therapeutic effect. Notably, tumor molecular characteristics, including PD‐L1 expression levels, tumor mutational burden (TMB), human leukocyte antigen diversity, and components of the tumor microenvironment, can contribute to substantial fluctuations in response rates, as shown in Table 3. Therefore, future studies need to further explore the association between these biomarkers and treatment efficacy and to accurately screen the advantaged population through multi‐omics analysis. Simultaneously, new biomarkers can be developed to monitor treatment effects in real time.

**TABLE 3:** Key biomarkers and clinical factors affecting the efficacy of combination therapy.

Influence factor	Effects on treatment outcomes	Clinical evidence
PD‐L1expression levels(TPS≥1%)	The ORR of patients with high expression was significantly improved	(KEYNOTE‐999)
TMB	Patients with higher TMB are more likely to activate systemic immune responses.	The response rate for melanoma patients increased to 65%
Timing of Radiotherapy (synchronous vs sequential)	The efficacy of simultaneous intervention (radiotherapy +ICIs 7–14 days) is better	The ORR for oligometastatic NSCLC improved to 58%
HLA diversity	The efficacy was limited in patients with low HLA‐I molecular diversity	The ORR of NSCLC with positive driver genes was only 18%
Immunosuppressive factors(TGF‐β)	Radiotherapy decreases the level of TGF‐β and enhances the effect of ICIs	Treg infiltration was reduced, and ORR increased after combination therapy

The application of biomarkers must extend beyond static assessments to dynamic monitoring during treatment. Liquid biopsies that analyze circulating tumor DNA (ctDNA) and immune cell subsets can provide real‐time insights into tumor evolution and treatment responses, thereby enabling adaptive therapy modifications. Furthermore, integrating spatial transcriptomics and multiplex immunohistochemistry will elucidate the functional state and geographical relationships of immune cells within the tumor microenvironment, thereby providing a more nuanced understanding of treatment success or failure.

## 5 CONCLUSION

The interaction between ICIs and radiotherapy is a major scientific breakthrough in cancer treatment. Through complementary mechanisms, these two approaches reshaped the TIME to substantially enhance antitumor responses. Although there are still many clinical challenges, with the continuous innovation of radiotherapy technology and the in‐depth exploration of new immune targets, combination therapy strategies are expected to become the core strategy of precision treatment for a variety of tumor types. Future research needs to integrate radiobiology with models of dynamic immune evolution, utilizing artificial intelligence algorithms to quantify the relationship between radiotherapy dose and immune activation (the functional relationship between STING pathway activity and fractionation dose^58^). It should also aim to construct a multidimensional response prediction system based on neoantigen load, T‐cell receptor repertoire diversity, and microenvironmental inhibitory factor profiles. This holds promise for achieving precise tumor therapy, thereby improving clinical treatment efficacy.

## CONFLICT OF INTEREST STATEMENT

All authors declare that they have no conflicts of interest regarding the publication of this manuscript. No financial or personal relationships with any organizations or companies that could inappropriately influence this work exist.
